# The prognostic factors of resected non-small cell lung cancer with chest wall invasion

**DOI:** 10.1186/1477-7819-10-9

**Published:** 2012-01-12

**Authors:** Chang Young Lee, Chun Sung Byun, Jin Gu Lee, Dae Joon Kim, Byoung Chul Cho, Kyung Young Chung, In Kyu Park

**Affiliations:** 1 Department of Thoracic & Cardiovascular Surgery, Seoul National University Hospital, Seoul National University College of Medicine; 2 Department of Internal Medicine, Yonsei University College of Medicine, Seoul National University Hospital, Seoul National University College of Medicine; 3 Department of Thoracic and Cardiovascular Surgery, Seoul National University Hospital, Seoul National University College of Medicine

**Keywords:** Chest wall, lung cancer, prognosis, adjuvant chemotherapy

## Abstract

**Background:**

We retrospectively reviewed the clinical features and surgical outcomes of patients with a surgically resected NSCLC invading chest wall in order to identify prognostic factors that impact long term survival.

**Methods:**

Between January 1990 and December 2009, 107 patients who underwent surgical resection for chest wall invading NSCLC were reviewed. Tumors invading only the parietal pleura were defined as superficial invasions, and those involving the soft tissue or ribs were defined as deep invasions.

**Results:**

There were 91 men and 16 women; median age was 64 years (range 30 to 80 years). Overall 5 year survival rate was 26.3%. The univariate prognostic factors for survival included gender, extent of resection (pneumonectomy vs lobectomy), tumor size(> 5 cm vs ≤ 5 cm), nodal status (N0 or N1 vs N2), completeness of resection (complete vs incomplete) and completeness of adjuvant chemotherapy. At multivariate analysis, five independent prognostic factors were shown; depth of invasion (superficial vs deep), tumor size, nodal status, completeness of resection, and completeness of adjuvant chemotherapy. In patients with completely resected T3N0 NSCLC, completion of chemotherapy is the only prognostic factor for long term survival.

**Conclusions:**

Completeness of resection, nodal status, depth of invasion, tumor size, and adjuvant chemotherapy were prognostic factors for long-term survival in NSCLC patients with chest wall invasion. Because of poor prognosis in cases with chest wall invasion that have N2 positive LN, that is difficult to achieve complete resection and that need pneumonectomy, definite chemoradiotherapy or neoadjuvant chemoradiotherapy should be considered first in these cases.

## Background

Although incomplete resection and the presence of nodal involvement, especially in the N2 station, have been consistently reported as poor prognostic factors of non-small cell lung cancer with chest wall invasion, other factors influencing survival are still unclear [1-11].

Whether adjuvant chemotherapy or radiotherapy is mandatory for patients with completely resected chest wall invading NSCLC without nodal involvement remains under debate [2,5,6,12,13].

Also, there are suggestions that the depth of chest wall invasion may influence prognosis following resection of lung cancer [11].

Herein, we retrospectively reviewed the clinical features and surgical outcomes of patients with a surgically resected NSCLC invading chest wall in order to identify prognostic factors that impact long term survival and to suggest the optimal treatment strategy according to these prognostic factors.

## Materials and methods

### Patients

Between January 1990 and December 2009, 107 patients with chest wall invading NSCLC were surgically treated at our institution. Patient records were obtained from a database that contained prospectively collected data cohort for any patient undergoing surgery for lung malignancies at our departments. Among 2,138 patients in our data cohort, 282 patients with pT3 NSCLC were selected. Patients with a T3 tumor in the invasion of mediastinal pleura, diaphragm, or the pericardium were excluded. Also, patients with tumor close to main bronchus (< 2 cm) were not included. Patients who received any type of preoperative chemotherapy or radiotherapy, or who underwent wedge resection or segmentectomy, were not selected. However, the subset of patients with T3 superior sulcus tumor included in this study had a chest wall invasion confined only to the parietal pleura or the soft tissue in thorax, which was completely removed through a single incision.

The charts and pathologic report of 107 patients were reviewed. Demographic data and first presenting symptoms were collected. The preoperative evaluation included a physical examination, routine blood tests, electrocardiography, pulmonary function test, and a lung perfusion scan if necessary. The routine staging workup included a chest computed tomography (CT) scan, abdomen CT scan or ultrasonography and fiberoptic bronchoscopy. Bone scintigraphy was routinely performed before 2007 in 104 patients. Fluorodeoxyglucose positron emission tomography (FDG-PET) was performed in 14 patients since 2003. Mediastinoscopy was selectively performed in 28 patients when it was suspected that the patient had enlarged mediastinal lymph nodes on the CT scan or PET scan. Cancers were staged according to the the 6^th ^edition of TNM Classification of Malignant Tumors.

### Surgical technique and pathologic examination

In all patients including those with a superior sulcus tumor except for one patient, posterolateral thoracotomy was performed. Systematic lymph node dissection was completed in all patients.

When chest wall invasion was preoperatively suspected, a chest wall resection including the affected rib was scheduled. Extrapleural resection was performed when the parietal pleura could be easily freed from the chest wall. In case of intraoperative evidence of deep parietal invasion, a decision on whether a chest wall resection or a wide extrapleural resection with involved soft tissue should be performed was determined according to each surgeon's decision. In these cases, resection margin was confirmed intraoperatively through the frozen section for complete resection. Pneumonectomy was performed whenever the mass crossed over the major fissure.

In a pathological examination of the resected specimens, tumors invading only the parietal pleura were defined as superficial invasions, and those involving the soft tissue or ribs were defined as deep invasions.

A complete (R0) resection was defined as a pathologic demonstration of negative tissue margins. Patients who had a complete gross resection in the thoracotomy but were found to have positive margins or extracapsular invasion of retrieved LN on the final pathologic review were classified as having undergone microscopically incomplete (R1) resections. A gross residual disease after attempted resection was classified as R2.

### Adjuvant treatment

There was no consistent institutional protocol with regard to adjuvant treatment for patient with surgically resected NSCLC invading the chest wall. Adjuvant treatment was determined by each surgeon and the referred oncologist. Three or more cycles of platinum based chemotherapy were regarded as a completion of chemotherapy. When radiotherapy was performed, it consisted of parietal radiotherapy in cases of stage IIB diseases, and parietal and mediastinal radiotherapy in cases of stage IIIA diseases.

### Recurrences and survival

A recurrence of the ipsilateral chest wall, lung, pleura, mediastinum, or cervical lymph node was defined as loco-regional recurrences. The remaining recurrences were defined as systemic recurrences.

Survival was calculated from the date of surgery until death or the date of the last follow-up. Survival rates, including postoperative and non-cancer related deaths, were calculated by the Kaplan-Meier method and compared using the log-rank test. Percentage comparisons were made by the χ^2 ^test. Univariate and multivariate analysis were performed using the Cox proportional hazards regression model to determine factors potentially predicting survival. Factors identified at p < 0.20 by univariate analysis and known prognostic factors such as age and radiotherapy were selected for inclusion in a multivariate Cox proportional hazards regression model. All statistical analyses were performed using the SPSS 12.0 software package (SPSS, Chicago, Illinois). Results were considered significant if the *p *value was less than 0.05.

## Results

### Demographics

There were 91 men and 16 women; the median age was 64 years (range 30 to 80 years). One or more of the following symptoms were present in the 96 patients: chest pain (n = 45, 41%), cough (n = 47, 44%), hemoptysis or blood tinged sputum (n = 19, 18%), and dyspnea (n = 7, 7%). Eleven patients (10%) were asymptomatic. Seventy one (66%) patients had a history of smoking. The mean FEV1 was 77% of the predicted value (range 22 to 133%). A superior sulcus tumor occurred in 17 (16%) patients.

### Surgical outcomes and pathologic examinations

Fifty five (54%) lobectomies, 4 (4%) bilobectomies and 45 (42%) pneumonectomies were performed. A chest wall resection or extrapleural resection were performed in 29 (27%) and 78 (73%) patients, respectively. After the chest wall resection, large chest wall defects were repaired using a prosthesis in 14 patients. The operative mortality was 5% (n = 5). Respiratory failure was the only cause of death. Perioperative complications occurred in 22 (21%) patients. There were 12 pulmonary complications such as pneumonia or atelectasis. Among them, 6 patients suffered from acute respiratory distress syndrome (ARDS) which needed mechanical ventilator care. Bronchopleural fistula (BPF) and bleeding necessitating re-exploration occurred in one patient, respectively.

The mean tumor diameter was 6.2 cm (range, 2 to 15 cm). There were 60 (56%) squamous cell carcinomas and 25 (23%) adenocarcinomas. In 67 (63%) patients, tumoral invasion was limited to parietal pleura; in 40 (37%) patients, it extended to the soft tissue or ribs. Pathologic stages were T3N0 in 64(60%) cases, T3N1 in 19 (18%) cases and T3N2 in 24 (22%) cases. Complete resection was achieved in 90 (84%) cases. Among R1 resection, there were 11 cases in the positive lateral resection margin and 5 cases in extracapsular invasion of retrieved lymph nodes. R2 resection occurred in 1 patient. Lymphovascular invasion was found in 18 patients. These results are summarized in Table 1.

**Table 1:** Demographic, surgical outcomes and pathologic features

	N = 107
Age (years, median (range))	64.0 (30-80)
Sex (male)	91 (85%)
FEV1 (liter, mean ± SD)	2.0 ± 0.8
FEV1 (%, mean ± SD)	76.5 ± 25.9
Superior sulcus tumor	17 (16%)
Extent of resection	
Lobectomy	58 (54%)
Bilobectomy	4 (4%)
Pneumonectomy	45 (42%)
Type of resection	
Chest wall resection	29 (27%)
Extrapleural resection	78 (73%)
Mortality	5 (5%)
Tumor size (mean ± SD)	6.2 ± 2.5
Depth of chest wall invasion	
Superficial invasion	67 (63%)
Deep invasion	40 (37%)
Nodal status	
N0	64 (60%)
N1	19 (18%)
N2	24 (22%)
Lymphovascular invasion	18 (17%)

FEV1; forced expiratory volume in 1 second

### Adjuvant treatments

Among the 102 patients who did not die perioperatively, fifty one patients were referred for adjuvant chemotherapy. However, only 35 (69%) patients completed adjuvant chemotherapy. In particular 19 patients (29.7%) out of 64 patients with pT3N0 received adjuvant chemotherapy. Eight patients refused chemotherapy after one or two cycles of chemotherapy. During the 1^st ^or 2^nd ^chemotherapy, systemic metastasis was found in 5 patients. Treatment-related toxicity, such as pneumonia or BPF, occurred in 3 patients, causing a halting of any further treatment. Chemotherapy related mortality occurred in one patient due to pneumonia and septic shock. Postoperative radiotherapy was carried out in 51 (50%) patients. Eighteen patients received postoperative chemoradiotherapy. No adjuvant treatment could be considered in 18 patients due to poor performance status and patient refusal.

### Recurrences and survivals

**Figure 1:**
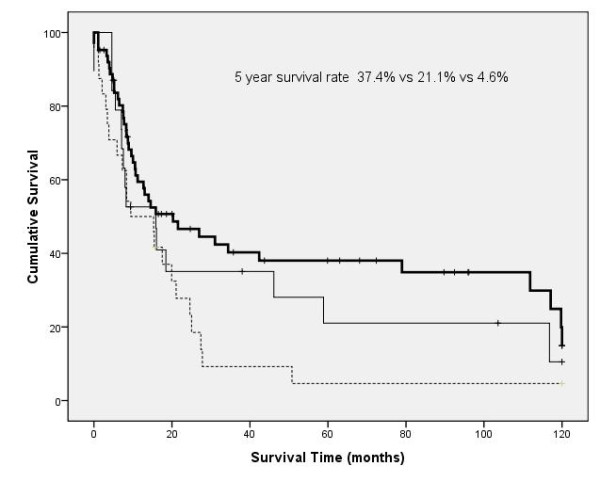
**Survival curve according to node involvement (thick solid line: N0, solid line: N1, dotted line: N2)**.

**Figure 2:**
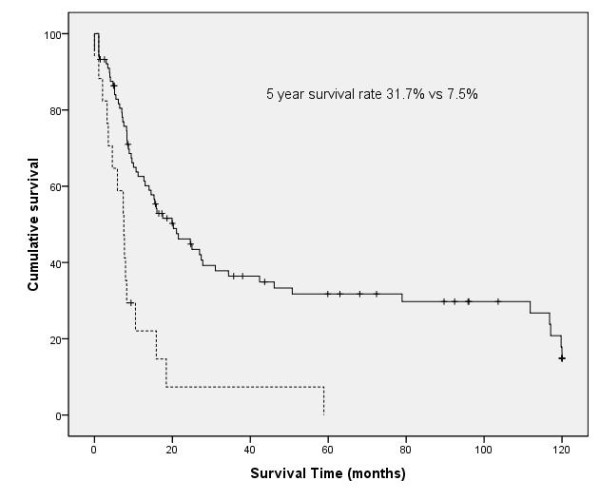
**Survival curve according to completion resection (solid line: complete resection, dotted line: non complete resection)**.

Median follow up time was 16 months. Recurrences occurred in 54 patients. Eleven patients had only loco-regional recurrences and 8 patients had loco-regional recurrences and systemic recurrences simultaneously. The locations of the systemic recurrences were as follows: Lung (n = 15), brain (n = 15), bone (n = 11), liver (n = 5), adrenal gland (n = 3) and others (n = 4). Twenty nine patients are currently alive. Out of 78 patients who were not alive during the follow-up period, 46 patients (59%) died within 1 year after the operation. The causes of 1 year mortality were as follows: cancer-related (n = 25, 54%), pneumonia or respiratory failure (n = 16, 35%), others (n = 3, 7%) and unknown (n = 2, 4%). One year mortality was higher in patients who underwent pneumonectomy compared to those who underwent lobectomy (57% vs 31%, *p *= 0.011). The median survival was 15.9 months and overall 5 year survival rate was 26.3%. According to the N status, 5 year survival was 37.4%, 21.1% and 4.6% in cases of N0, N1, and N2 disease, respectively (*p *= 0.047, Figure 1). The 5 year survival rate of patients with complete resection (31.7%) was statistically higher than those with incomplete resection (7.5%, p < 0.001, Figure 2). The univariate prognostic factors for survival included gender (*p *= 0.029), extent of resection (pneumonectomy vs lobectomy, *p *= 0.041), tumor size(> 5 cm vs ≤ 5 cm, *p *= 0.001), nodal status (N0 or N1 vs N2, *p *= 0.029), completeness of resection (complete vs incomplete, *p *< 0.001) and completeness of adjuvant chemotherapy (*p *< 0.001). At multivariate analysis, five independent prognostic factors were shown; depth of invasion (superficial vs deep, *p *= 0.035), tumor size (*p *= 0.007), nodal status (*p *= 0.001), completeness of resection (*p *< 0.001), and completeness of adjuvant chemotherapy (*p *< 0.001, Table 2).

**Table 2:** Univariate and multivariate analysis for long term survivals

	Univariate analysis				Multivariate analysis			
	*p *value	Odds ratio	95% CI		*p *value	Odds ratio	95% CI	
						
			lower	upper			lower	upper
Sex (male/female)	0.029	2.10	1.01	4.37	0.270	1.59	0.70	3.61
Age(≤ 65/> 65))	0.278	0.78	0.50	1.22	NA	NA	NA	NA
Extent of resection (pneumonectomy/lobectomy)	0.041	1.59	1.02	2.49	0.410	1.24	0.75	2.06
Type of resection (Extrapleural resection vs chest wall resection)	0.145	0.70	0.43	1.13	0.257	1.42	0.77	2.62
Tumor size(≤ 5 cm/> 5 cm)	0.001	0.44	0.27	0.71	0.007	0.46	0.27	0.81
Lymphovascular invasion (no/yes)	0.151	0.66	0.38	1.16	0.126	0.62	0.34	1.14
N2 lymph node involvement (N0,1/N2)	0.029	0.57	0.35	0.94	0.000	0.34	0.19	0.61
R0resection (R0/R1,2)	0.000	0.32	0.18	0.56	0.000	0.26	0.13	0.49
Depth of invasion (superficial/deep)	0.148	0.72	0.46	1.12	0.035	0.56	0.33	0.96
Superior sulcus tumor (no/yes)	0.939	1.02	0.58	1.83	NA	NA	NA	NA
Completion of chemotherapy (no/yes)	0.000	3.18	1.86	5.41	0.000	4.83	2.47	9.43
Radiotherapy (no/yes)	0.618	0.89	0.57	1.40	NA	NA	NA	NA

NA; not available

**Figure 3:**
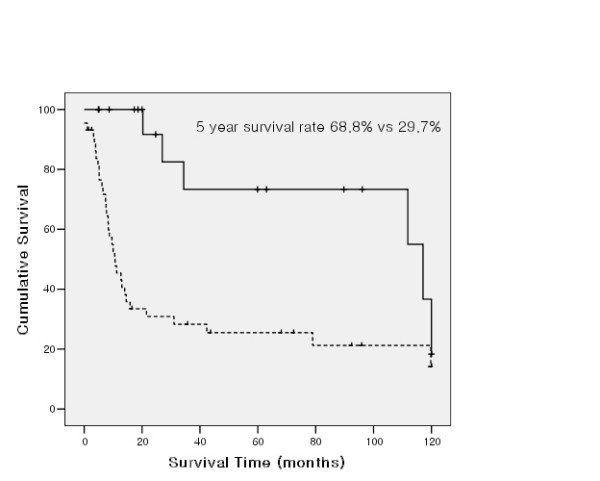
**Survival curve according to completion of chemotherapy in patients with completely resected T3N0 NSCLC (solid line: completion group, dotted line: non completion group)**.

5 year overall survival rates of patients with completion of adjuvant chemotherapy were statistically higher than those of patients without completion of adjuvant chemotherapy (5 year survival rate 46.7% vs 16.6%). Statistical analysis for cancer-specific survival showed that the survival benefit was higher in the patient groups who received chemotherapy (5 year survival rate 45.8% vs 21.8%, *p *= 0.001). And in patients with completely resected T3N0 NSCLC, the completion of chemotherapy is the only prognostic factor for long term survival (5 year survival rate 68.8% vs 29.7% *p *= 0.011, Figure 3) in univariate analysis. Our study also revealed that there was no statistical difference in survival with or without adjuvant radiotherapy (5 year survival rate 23.8% vs 29.9%, *p *= 0.618).

## Discussion

This study demonstrated that the long-term survival of patients with chest wall invading NSCLC were independently related to not only nodal status and R0 resection which have been consistently reported as prognostic factors, but also the depth of invasion, tumor size and adjuvant chemotherapy.

Since technical feasibility and the long-term survival of the surgically resected NSCLC invading chest wall were reported in 1947 [14], surgical resection has been the key component in the management of most patients with chest wall invading NSCLC. Except for the incompleteness of resection and the presence of lymph node metastasis, other factors influencing on survival are still unclear. In particular, controversies remain as to how extensive a resection should be [7-9,15,16] and on the role chemotherapy or radiotherapy [5,6,12,13].

Mean FEV1 of the patients enrolled in this study (76.5%) was significantly lower than that of the patients in the total cohort (92.1%) and this is thought to be the reason for high incidence of death due to pneumonia during the follow up period.

We performed a pneumonectomy in cases of large tumor that invaded to fissure and adjacent lobe. Relatively high percentage of pneumonectomy (42%) was performed compared to previous studies that reported percentage of 20-27% [7,8,10]. In the patients with tuberculosis and other inflammatory lung disease which were frequent in Korea, pneumonectomy was performed in cases large tumor crossed over major fissure and remnant lung volume is not enough. That's the reason why the incidence of pneumonectomy was high. Larger tumor size and high incidence of respiratory related mortality seemed to be attributable to significantly higher 1-year mortality of the patients who received pneumonectomy. Hence, pneumonectomy should be avoided in patients with chest wall invasion.

As previously reported [7-9], R0 resection and the absence of N2 disease was revealed as better prognostic factors for long term survival of patients with chest wall invading NSCLC in this study.

The prognostic significance of the degree of the chest wall invasion was not reflected in the 7^th ^TNM classification but the sub-classification of the T3 stage and prospective data collection were recommended for future revision [17]. There are several studies [1,7,8] including ours, that report the better prognosis of a patient with tumor invasion in the parietal pleura compared to deep invasion. In case of superficial invasion, extrapleural resection is thought to be an adequate treatment option, considering the fact that there were no significant differences in the 5-year survival between the patients with parietal pleural invasion (N = 67), who received extra-pleural resection (N = 57) and chest wall resection (N = 10) (29.7% vs 23.3%, p = 0.832), and the high incidence of complications among the patients who received chest wall resection [8,11]. In cases of deep invasion, moreover, there are no statistical differences between group of extrapleural resection and group of chest wall resection in terms of locoregional recurrence rates and 5 year overall survival rates if R0 resection was achieved.

Unlike the 6^th ^edition of the TNM staging, the T staging was sub-classified as 3 cm, 5 cm and 7 cm according to the tumor size in the 7^th ^edition of the TNM staging [17], and what it implicates is that the tumor size is an independent prognostic factor of NSCLC. The results of our study also show that the tumor size independently influences the prognosis of the NSCLC patient with chest wall invasion similar to the in other study [10].

The role of adjuvant chemotherapy in patients with completely resected NSCLC has been evaluated in many randomized clinical trials and meta-analysis. Cancer Care Ontario and American Society of Clinical Oncology concluded that adjuvant cisplatin-based chemotherapy is recommended for routine use in patients with stages IIA, IIB according to the 6^th ^edition of TNM Classification of Malignant Tumors [12]. However, De Pas et al [13] have asserted that the efficacy of adjuvant chemotherapy should be assessed separately in the T1-2N1 and T3N0 subgroups considering the fact that the efficacy of adjuvant chemotherapy usually varies according to lymph node status [14] and differences in biological behavior between subgroups. And they recommended that a pooled-analysis of the existing data would quickly and with limited effort provide a preliminary answer. Our result demonstrated that systemic recurrence occurred in about 80% of patients whom recurrences had occurred in, and adjuvant chemotherapy was also effective in terms of long-term survival especially in completely resected T3N0 patients. The cancer- specific survival was calculated because there was a subset of patients who could not receive chemotherapy due to their poor performance status and died of reasons such as pneumonia. Statistical analysis showed that the survival benefit was higher in the patient groups who received chemotherapy. These results implicate the possible benefit for postoperative chemotherapy for NSCLC patients with chest wall invasion although all confounding comorbidities were not completely reflected in cancer-specific survival. However, the fact that only 35 patients out of 107 patients, enrolled in this study, completed chemotherapy suggests the likelihood of preoperative chemotherapy in terms of expansion of delivery rate in definite preoperative chest wall invading T3 patients and the necessity of research in this area.

The role of adjuvant radiotherapy in patients with chest wall invading NSCLC is still controversial. Although two reports [15,18] about beneficial effect of adjuvant radiotherapy have been published, other series [2,5,7,19] have not showed any improvement in survival with use of postoperative radiotherapy. Our study also revealed that there was no statistical difference in survival with or without adjuvant radiotherapy.

The proposed strategy based upon prognostic factors mentioned above are as follow: Mediastinoscopy should be done irrespective of the chest CT or PET-CT results and neoadjuvant chemotherapy should be initiated if N2 is confirmed. Neoadjuvant chemoradiotherapy should be considered if the resection margins are expected to be insufficient due to large tumor size or deep invasion in the posterior location like Pancoast tumor [20,21]. Pneumonectomy should be avoided whenever possible because not only cancer related death but also respiratory related death is significantly high in this group of patients. If the tumor invades only the parietal pleura and separates without any remaining tumor tissue, an extrapleural resection could be done. Otherwise, chest wall resection should be performed to secure the wide resection margin. Postoperative adjuvant chemotherapy could be considered in patients with completely resected T3N0 chest wall invading tumors.

### Limitations

A small number of patients and the retrospective method of reviewing the data remain as limitations of the study. The same protocol was not applied because the data were collected from a long period of time. However, mediastinal lymph node dissection was performed and a platinum based chemotherapy agent was used in all patients and hence, treatment strategy was relatively consistent.

Patients with a superior sulcus tumor were included in this study, unlike many other studies, that exclude them due to different behaviors of the disease itself. However, patients with a superior sulcus tumor invading the structure beyond the thorax were excluded in this study and the influence of the included patients with superior sulcus tumor might be small because the survival differences between patients with superior sulcus tumor and those with non superior sulcus tumor were insignificant.

Long term survival benefit of adjuvant chemotherapy after lung cancer surgery is known to be approximately 5%, whereas the 5 year survival benefit in this study exceeded 30%. This result seems to be the influence of patients whose postoperative performance was poor, included in the group of patients who did not receive chemotherapy. Hence, we calculated the cancer-specific survival rate but we think that this was not enough to completely exclude the effect of selection bias as well. Accordingly, this study has a hypothesis generating effect regarding the role of chemotherapy in lung cancer patients with chest wall invasion.

## Conclusion

Completeness of resection, nodal status, depth of invasion, tumor size, and adjuvant chemotherapy were prognostic factors for long-term survival in NSCLC patients with chest wall invasion. Because of poor prognosis in cases with chest wall invasion that have N2 positive LN, that is difficult to achieve complete resection and that need pneumonectomy, definite chemoradiotherapy or neoadjuvant chemoradiotherapy should be considered first in these cases. Further verification is needed through a larger group study due to the small sample size and retrospectively reviewed data.

## Competing interests

Drs. Lee CY, Byun CS, Lee JG, Kim DJ, Cho BC, Chung KY and Park IK have no conflicts of interest or financial ties to disclose.

## Authors' contributions

CYL: Conception and design, drafting the article, final revision, CSB: Acquisition of data, interpretation of data, JGL: Revision for important content, DJK: Interpretation of data, revision for important content, BCC: Interpretation of data, revision for important content, KYC: Acquisition of data, interpretation of data, revision, IKP: Conception and design, revision, final approval. All authors read and approved the final manuscript.
